# Accuracy of cDNA microarray methods to detect small gene expression changes induced by neuregulin on breast epithelial cells

**DOI:** 10.1186/1471-2105-5-99

**Published:** 2004-07-23

**Authors:** Bin Yao, Sanjay N Rakhade, Qunfang Li, Sharlin Ahmed, Raul Krauss, Sorin Draghici, Jeffrey A Loeb

**Affiliations:** 1 Center for Molecular Medicine and Genetics, Wayne State University, Detroit, Michigan, USA; 2 Department of Neurology, Wayne State University, Detroit, Michigan, USA; 3 Department of Computer Science, Wayne State University, Detroit, Michigan, USA; 4 Newton, Massachusetts, USA; 5 MCBI Wayne State Node, Wayne State University, Detroit, Michigan, USA

## Abstract

**Background:**

cDNA microarrays are a powerful means to screen for biologically relevant gene expression changes, but are often limited by their ability to detect small changes accurately due to "noise" from random and systematic errors. While experimental designs and statistical analysis methods have been proposed to reduce these errors, few studies have tested their accuracy and ability to identify small, but biologically important, changes. Here, we have compared two cDNA microarray experimental design methods with northern blot confirmation to reveal changes in gene expression that could contribute to the early antiproliferative effects of neuregulin on MCF10AT human breast epithelial cells.

**Results:**

We performed parallel experiments on identical samples using a dye-swap design with ANOVA and an experimental design that excludes systematic biases by "correcting" experimental/control hybridization ratios with control/control hybridizations on a spot-by-spot basis. We refer to this approach as the "control correction method" (CCM). Using replicate arrays, we identified a decrease in proliferation genes and an increase in differentiation genes. Using an arbitrary cut-off of 1.7-fold and p values <0.05, we identified a total of 32 differentially expressed genes, 9 with the dye-swap method, 18 with the CCM, and 5 genes with both methods. 23 of these 32 genes were subsequently verified by northern blotting. Most of these were <2-fold changes. While the dye-swap method (using either ANOVA or Bayesian analysis) detected a smaller number of genes (14–16) compared to the CCM (46), it was more accurate (89–92% vs. 75%). Compared to the northern blot results, for most genes, the microarray results underestimated the fold change, implicating the importance of detecting these small changes.

**Conclusions:**

We validated two experimental design paradigms for cDNA microarray experiments capable of detecting small (<2-fold) changes in gene expression with excellent fidelity that revealed potentially important genes associated with the anti-proliferative effects of neuregulin on MCF10AT breast epithelial cells.

## Background

Spotted cDNA microarrays are used in high-throughput experiments that interrogate the relative expression of thousands of genes simultaneously for many biological processes with wide applications in biological and medical research. Typically in a two-dye spotted cDNA microarray experiment, two mRNA samples are transcribed into cDNAs, labeled with two different fluorescent dyes, commonly Cy3 and Cy5, and hybridized on the same slide. The relative gene expression level is then measured as a ratio of the intensities of the fluorescent dyes. However, the signal intensity of the dye, which indirectly represents the gene expression level, can be affected by many other sources of error such as dye efficiency, sample preparation, and the variability of the biological samples [1,2].

An important question is how to identify differentially expressed genes, some of which change only minimally (<2-fold), given many known and potentially unknown sources of variance in the microarray experiment. In order to reduce false positive rates, many published experiments use a cut-off of 2- to 3-fold [3-5]. This limits the ability of the microarray experiment to detect small, but biologically important changes. In fact, recent reports have shown that microarrays can significantly underestimate gene expression changes and therefore a high cut-off will miss important changes [6]. Although more sophisticated statistical methods have been proposed for single slide analysis [7-13], it is becoming clear that in order to reduce random variance, replication becomes more and more important in microarray experimental design by greatly increasing the power of the experiment to measure small gene expression changes [2,13-17]. As a relatively new technique, many new theories have been developed for data analysis and experimental design, but few of these theories have been rigorously tested against a well-established standard method such as the Northern blot.

In this paper we compared two experimental design and analysis methods performed on quadruplicate arrays that include a dye-swap design [18,19] and a modified reference design method that uses a control-control hybridization to correct for systematic experimental errors, that we refer to as the "control correction method" (CCM). We demonstrate that both experimental designs accurately identified small (<2-fold) gene expression changes after a 24-hour treatment of MCF10AT breast epithelial cells with the growth and differentiation factor neuregulin. These changes correlate well with the anti-proliferative effects of neuregulin resulting in a relative decrease in proliferative genes and increase in anti-proliferative genes that will be important for future investigations.

## Results

**Figure 1:**
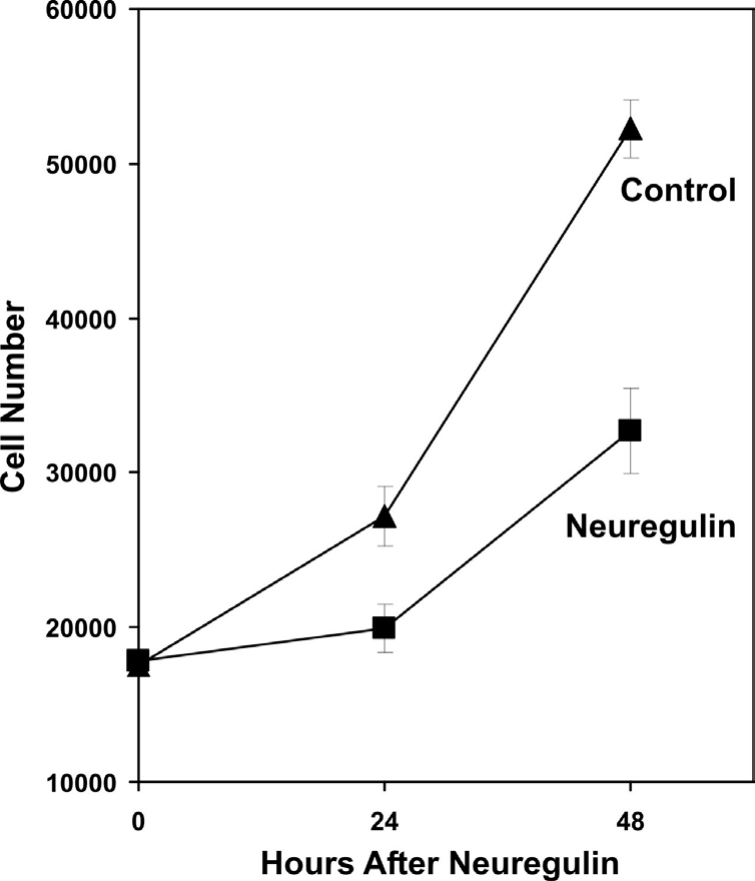
Anti-proliferative effects of neuregulin on MCF10AT cells. Quadruplicate cultures of MCF10AT cells were treated with and without 1 nM neuregulin 3 days after plating and cell counts were performed demonstrating a significant decrease in their growth 24 and 48 hours after treatment. The p value for 24 hr was 0.0011, and for 48 hr was 1.46E-05.

The results presented in this paper demonstrate two, complementary cDNA microarray methods capable of reliably revealing small changes in gene expression in transformed human breast epithelial MCF10AT cells after treatment with neuregulin. Since, as shown in Fig. 1, treatment of these cells with neuregulin significantly slows their growth rate, identifying early gene expression changes in this process will be important in understanding how neuregulin regulates cell growth in both normal and malignant breast epithelium, and will also provide both biological markers and potential targets in breast cancer. Large quantities of highly purified total RNA were isolated from MCF10AT cells treated with or without neuregulin for 24 hours and used both for microarray experiments and northern blot confirmation studies.

### Experimental designs to address systematic errors

**Figure 2:**
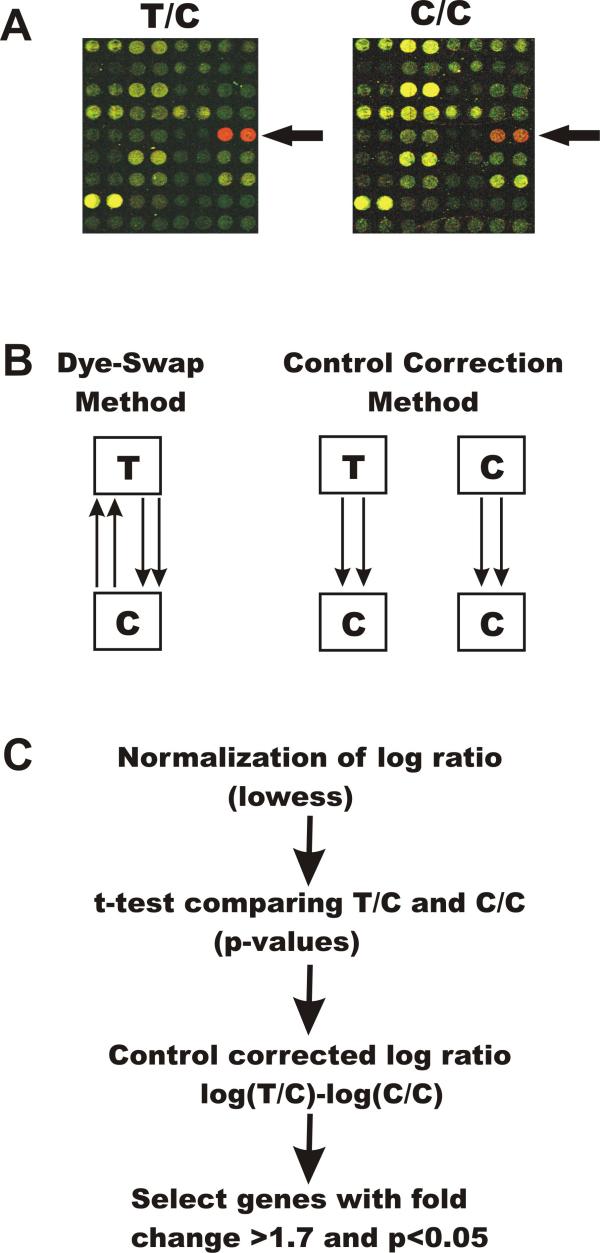
(**A**) Highly reproducible systematic errors from gene-dye interactions. The arrows demonstrate intense red dye labeling for a given gene spotted in duplicate both for the T/C slide where the treated sample is labeled with red dye (Cy5) and control sample is labeled with green dye (Cy3), as well as in the C/C slide where the same sample is labeled both red and green. (**B**) Experimental designs. Two different experimental methods were compared: A dye-swap approach, where the dye color is reversed for T/C hybridizations, and a "control correction" design, where T/C and C/C hybridizations are performed without reversing the dyes. T denotes the neuregulin treated cells, while C denotes the untreated, control cells. Each arrows represent a replicate and the tails of the arrows indicate cy5 labeling and the heads indicate cy3 labeling. (**C**) Data processing flow chart for the control correction method.

As with most experimental methods, replicate measurements can reduce random errors. Equally important are systematic errors. Systematic errors result from a constant tendency to over- and under-estimate true values and cannot be eliminated by replicate analysis, since they are often highly reproducible. An example of such a systematic error is a gene-specific dye effect, also called "dye–gene" interaction [18], and is shown in Fig. 2A. For a given gene spotted in duplicate (arrows), the red signal labeling the treated sample (T) is much brighter than the green signal for the control sample (C). This was highly reproducible for both spots on the same array and between multiple arrays. One way to determine whether the apparent up-regulation of this gene is true, is to use the same control sample labeled with both red and green dyes and perform a control/control (C/C) hybridization. Fig. 2A shows that the same intense red signal is seen in the C/C hybridization as was seen in the treated/control (T/C), demonstrating that this signal is a systematic error producing a false positive gene expression change.

Given the unavoidable presence of these systematic errors, methods to correct these errors are needed. One way to correct for systematic errors in microarray experiments is to take advantage of C/C hybridizations to correct the T/C hybridizations. This requires a modified reference design, which we refer to as a "control correction" design. This is different from a common reference design used previously [19,20]. Here, each spot of the T/C hybridization is "corrected" by the same spot from the C/C hybridization for systematic errors. A second method that will also correct for systematic errors is a "dye-swap" design [16,17,19]. The dye-swap design uses an ANOVA to calculate gene expression changes from replicate cDNA microarrays probed with T/C hybridizations performed where the dye color is swapped. Included in the ANOVA are factors to correct for systematic errors such as dye and dye-gene interactions. The "control correction" and the dye-swap designs are compared in Fig. 2B. Each of these experimental designs was performed on quadruplicate arrays. Each of these two designs required its own analysis method. While we used an analysis method that utilizes individual t-tests for each spot for the CCM, we compared both ANOVA and Bayesian analysis methods for the dye-swap design.

### Control correction method experimental design and results

**Figure 3:**
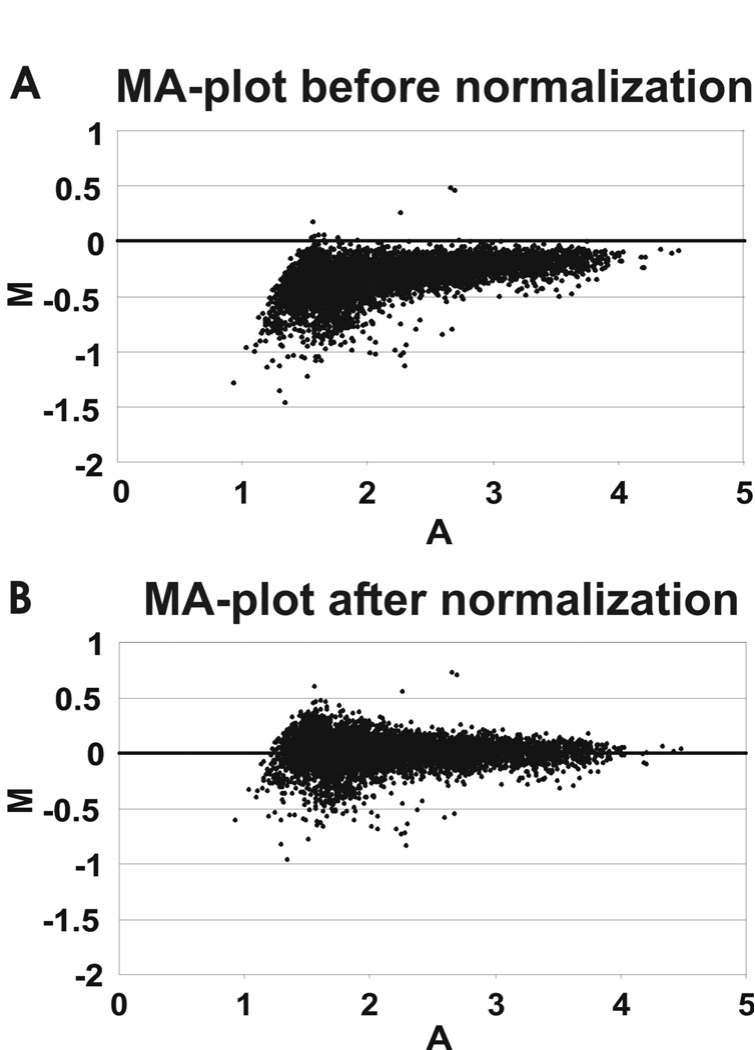
Array and intensity-dependent variation can be corrected by normalization based on intensity. (**A**) This is an MA-plot before normalization for one of T/C slides that plots the log intensity ratios against the averaged intensities at both wavelengths: M = log (T/C) and A = 1/2log(T*C). The majority of the data is less than zero in a "banana" or "comma" shaped distribution. This demonstrates a systematic, intensity-dependent dye effect, prominent at lower intensities. (**B**) After normalization using the lowess function, the MA-plot shows a more even distribution at all intensities.

A flow chart for the control correction method is shown in Fig. 2C. All microarrays used in this study were from the same lot of 3333 gene spotted cDNA slides (similar to the commercially available NEN MicroMax 2400 slides with 933 additional genes (Alphagene Inc., Woburn, MA) where each gene was spotted in duplicate, and hybridized using an optimized, two-step hybridization protocol with either Cy3 or Cy5-labeled dendrimer complexes (Genisphere, Hatfield, PA). A key advantage of the Genisphere Dendrimer system is the need for only 3 μg of total RNA per array without the need for a potentially non-linear amplification step to boost the signal. After scanning and spot-wise local background correction (Imagene Software, Biodiscovery, CA), a log Cy5/Cy3 ratio versus log signal intensity MA plot was prepared and shown in Fig 3A[20]. Without any correction, the ratio vs. intensity plot shows a banana shape as ratios trend downward in the low intensity range. This suggests an intensity-dependent dye effect. In order to correct this and to normalize data sets between different slides, an intensity-dependent normalization procedure was performed that fits the data to a lowess curve as a function of signal intensity [21]. After normalization, the log ratios became more evenly distributed around zero (Fig. 3B).

**Figure 4:**
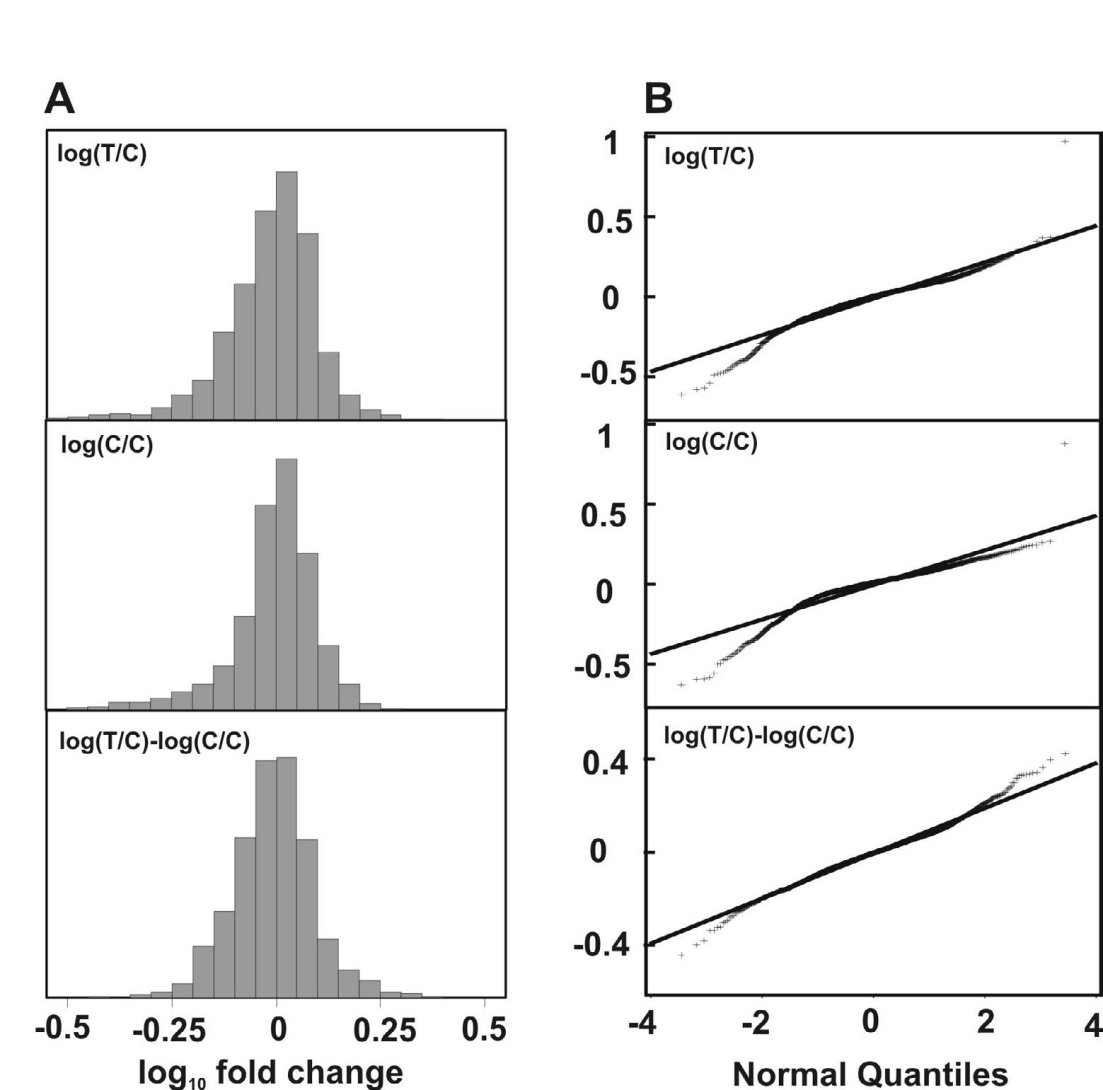
Control correction of each spot markedly improves the distribution of log ratios. (**A**) Histograms show that T/C and C/C log ratio distributions after lowess normalization still have a marked asymmetry with a larger tail towards the left (increased down-regulated genes). The distribution becomes symmetric after subtracting the log (C/C) from the log (T/C). (**B**) Quantile-quantile plots similarly show that the log ratio distribution becomes more normal after correction of each spot with the control ratio.

However, despite this relatively even distribution, histograms of normalized log ratios for T/C and C/C display long tails to the left as shown in the histograms in Fig. 4A and the quantile-qauntile plots in Fig. 4B. Since there should be no treatment effects on the C/C slides, a symmetric, normal distribution would have been expected. The skewed appearances of the normalized distributions indicate additional, uncorrected systematic errors in both T/C and C/C hybridizations. "Correction" of each spot by subtracting the log (C/C) ratios from the log (T/C) ratios produces an approximately normal distribution of the log (T/C) ratios (shown on the bottom of Figs. 4A and 4B). In addition to the systematic errors that occur on a spot-by-spot basis shown in Fig. 2A, systematic errors were found as a function of slide location, particularly at the edge of the arrays. These errors were also corrected by this method (data not shown). Yang and Dudoit proposed a within slide normalization for this type of spatial effect [21], however, one concern for within slide normalization is that if the number of genes is small in each spatial group, the assumption that there will be an equal proportion of up- and down-regulated genes may be untrue.

**Figure 5:**
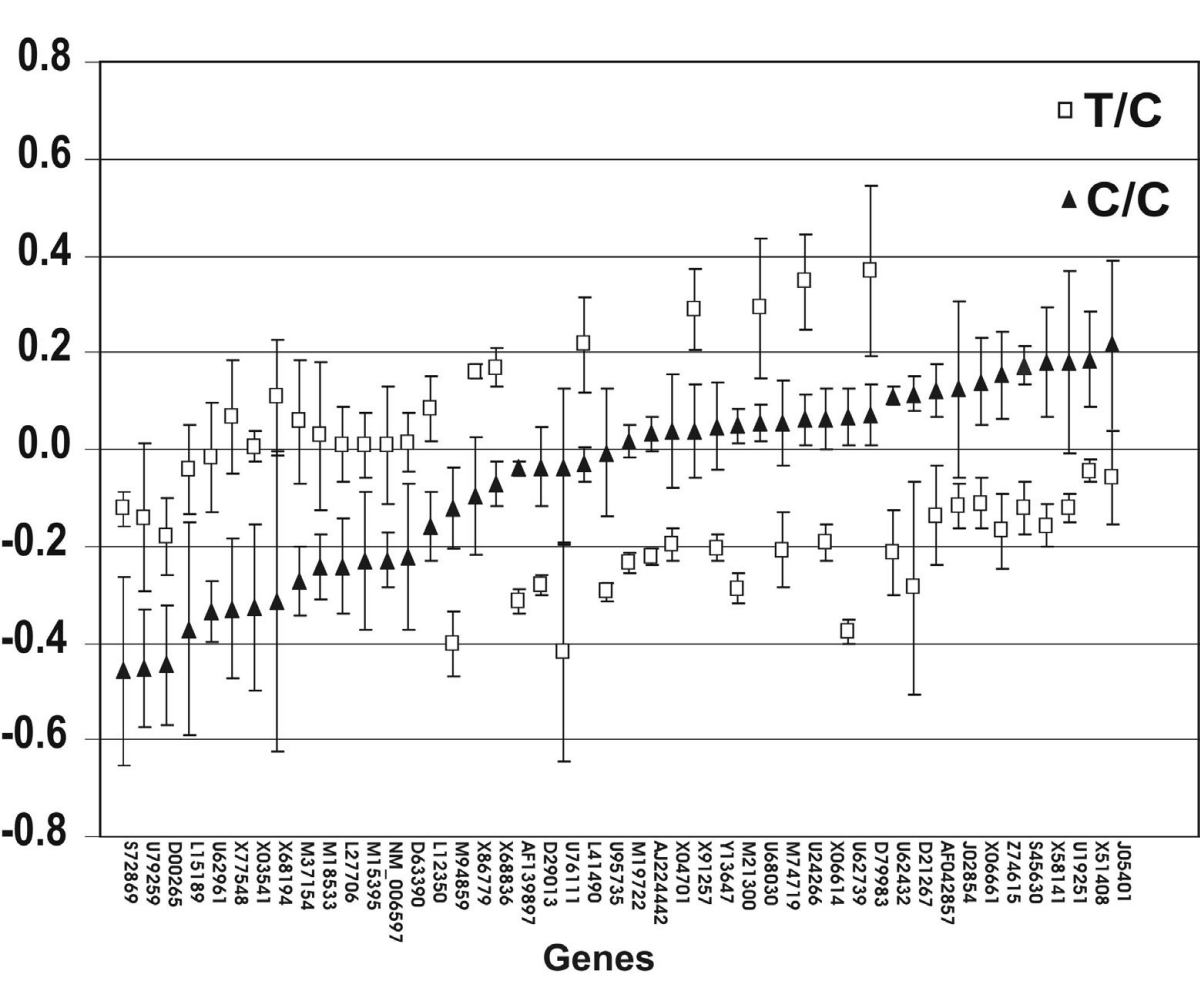
The control correction method identifies gene expression changes from spots with variable C/C ratios. The log ratios for each gene are plotted both for the T/C (□) and the corresponding C/C (▲) hybridizations for the 46 genes selected from control correction method. The error bars represent one standard deviation in each direction.

**Figure 6:**
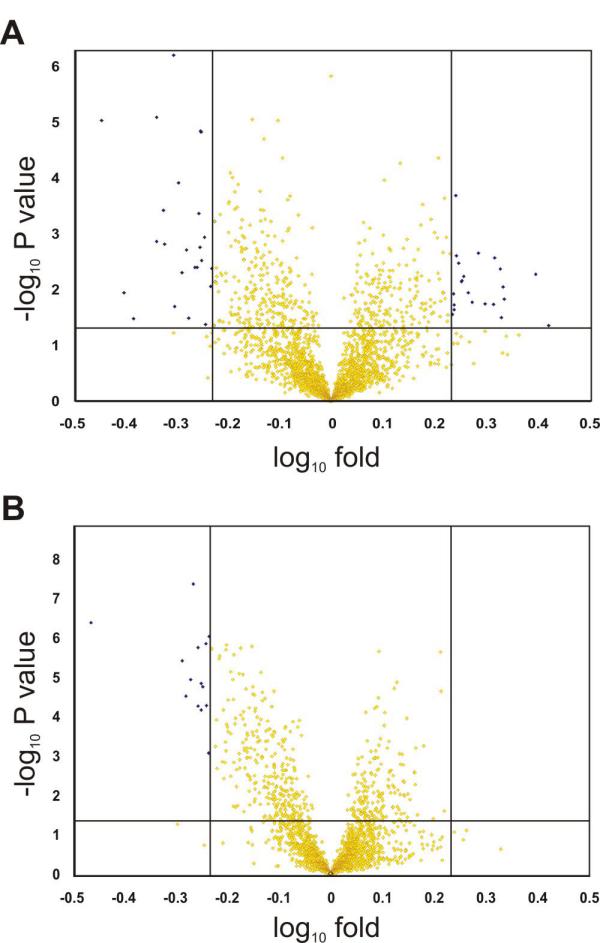
Both control correction and dye-swap methods reveal statistically significant changes in gene expression. Volcano plots of the control correction method (**A**) and the dye-swap method (**B**) reveal a small proportion of genes that met our arbitrary criteria of having >1.7 fold changes with p values <0.05, determined individually for each gene. The horizontal lines on each graph represent p = 0.05. The vertical lines represent 1.7 fold changes, both up- and down-regulated. Genes shown in blue in upper left and right areas were selected for northern blot confirmation.

As a final step, a t-test was performed to compare the normalized log ratios of T/C and C/C for each gene. This yields p values for each control-corrected fold change calculated as log (T/C)-log (C/C). In Fig. 5, the average and standard deviation of gene expression ratios for the log (T/C) and log (C/C) are plotted for the genes using 1.7-fold and p < 0.05 cut-offs. This clearly demonstrates the importance of correcting each log (T/C) value with the corresponding log (C/C) control value. For example, while some log (T/C) ratios are close to zero, by using the log (C/C) as baseline, true gene expression changes above or below this were identified that would otherwise have been missed. The 1.7-fold cutoff was chosen to be within the detection range of northern blot analyses, which we felt would be the most sensitive method to confirm these small changes. A volcano plot, shown in Fig. 6A, summaries 46 differentially-regulated genes that met these criteria for the CCM.

### Comparison of the control correction to the dye-swap design

Many have proposed that a dye-swap experimental design combined with an ANOVA will correct for systematic errors [17-19]. To verify this and compare the dye-swap design to the control correction design, a dye-swap experiment was performed on quadruplicate arrays using the same RNA samples and the two interconnect ANOVA model of Wolfinger et al [22,23]. Using this experimental design with the same cut-off values, 14 differentially expressed genes were identified and are presented as a volcano plot alongside that of the CCM (Fig. 6B).

Table 1 lists those genes that met our selection criteria, together with their fold-change, p values, and functional classifications. Only 5 genes were found in common for both methods. The genes have been broadly grouped into proliferation, differentiation, and unclassified genes in order to observe trends in the neuregulin-induced gene expression changes that could be important in regulating cell growth. A general trend showing a down-regulation of proliferation genes and up-regulation of differentiation genes was observed. This includes several oncogenes, cell cycle control and cell proliferation genes that were all down-regulated; and tumor suppressor genes, growth inhibition and differentiation genes were up-regulated. This pattern is consistent with the anti-proliferative/differentiation effects of neuregulin on MCF10AT human breast epithelial cells.

### Verification of microarray accuracy by northern blot analysis

**Figure 7:**
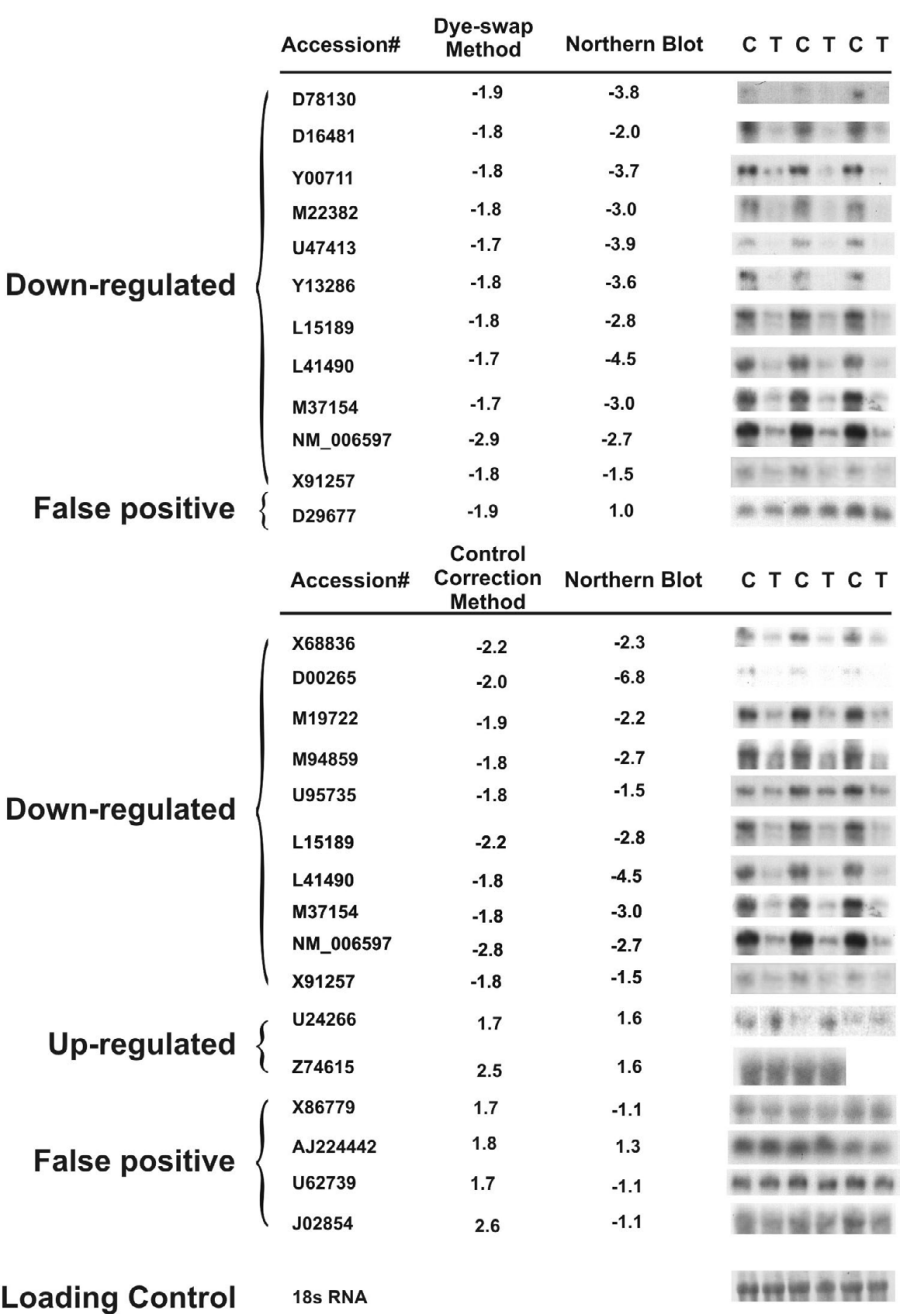
Northern blots confirm a majority of gene expression changes for both methods. The far right-hand column shows northern blot results performed in triplicate for genes identified by each microarray design method. For each gene, the fold-change from the microarray together with the average fold-change quantified from the northern blots is shown. To correct for loading differences, in the northern blots each measurement was normalized to the corresponding amount of 18S rRNA measured on each gel. A representative example of the 18S rRNA is shown on the bottom of the figure. Two of the Z74615 northern blot bands were discarded due to contamination. Down-regulated, up-regulated, and false positive genes that were not confirmed by northern blots are indicated.

To confirm these gene expression changes and to determine the accuracy of each experimental method, we selected 23 genes for verification by northern blot. We chose all 5 genes detected by both methods, 6 up-regulated and 5 down-regulated genes from the control correction design, and 7 genes from the dye-swap experiment. The selection of genes was not random, as we selected a balanced complement of genes of variable intensity that were both up- and down-regulated. The probes used for northern blots were generated by PCR from clones used to spot the arrays. Each blot contained triplicate control and treated samples and was re-probed multiple times. Fig. 7 summarizes the northern blot results for these 23 genes. The band intensities were quantified, normalized to total ribosomal RNA for each gel, and averaged to produce a fold change that was compared directly to the fold change from the microarrays. In general, differential gene expression was confirmed by the northern blots for both array design methods. For the dye-swap method only 1 of 12 genes was a false positive, while 4 out of 16 genes were false positives in the control correction method. Down-regulated genes were verified more reliably in the control correction method (10/10) than up-regulated genes (2/6). All differentially expressed genes common to both methods were confirmed..

Since the ANOVA method we used can sometimes underestimate the variance, we re-analyzed our dye-swap data with a Bayesian method using a regularized t-test as implemented in Cyber-T [24]. This analysis revealed 16 differentially expressed genes using the same cut-offs, 10 of which were in common with the ANOVA method (Table 1). A greater number of genes were identified using the regularized t-test, and the corresponding p values for these genes were lower. Based on the previous northern blot data, 8/9 (89%) of these were confirmed.

## Discussion

### Gene expression changes in MCF10AT cells suggest a rapid anti-proliferative effect of neuregulin

MCF10AT cells are a human breast epithelial cell line stably transfected with a mutant ras oncogene. These cells are pre-malignant, but can progress to invasive carcinoma [25,26]. Given that neuregulin can differentially affect the growth properties of different cell lines, we used the MCF10AT cell line as model system to identify genes that may be down-stream from neuregulin activation and could thus be studied further for their roles in breast cancer cells that respond differentially to neuregulin. Combining two cDNA microarray experimental design methods, we have identified genes differentially expressed by neuregulin treatment that correlated with a significant decrease in their growth rate. The pattern of expression clearly shows an anti-proliferative effect of neuregulin on the MCF10AT cells with a reduction in genes associated with proliferation such as heat shock proteins, oncogenes, cell cycle control genes, genes involved in fatty acid and sugar synthesis, transcription and translation together with an increase in differentiation genes including tumor suppressor genes, DNA damage repair genes, growth inhibition genes and differentiation genes. We further showed that these effects are biologically consistent with the rapid, anti-proliferative effects of neuregulin on cell number. Additional experiments have shown that these genes are important biological markers for the degree of malignancy in other breast epithelial cell lines that have differential proliferation responses to neuregulin (Li Q, Ahmed S, and Loeb JA, unpublished results).

### Both experimental designs demonstrate a high confirmation rate for small changes in gene expression

One of the important tasks in microarray technology is to design experiments and develop statistical tools to obtain data efficiently and accurately to answer fundamental questions in biology. In many experiments, this requires the ability to detect small changes in gene expression with high fidelity. In this study we compared two common experimental design paradigms for cDNA microarrays and determined their accuracy by northern blot. Both methods identified small expression changes with considerable accuracy. In the control correction design, we used control hybridizations to correct for systematic errors on a spot-by-spot basis. The method is based on an assumption that systematic errors from slides made from the same lot and processed identically do not vary significantly. To minimize the possible variance of systematic errors in T/C slides and C/C slides we maintained strict experimental conditions, such as same-day sample preparation and same-day hybridization. We also used the same control samples for both the T/C and C/C hybridizations instead of using an arbitrary control sample that might be quite different in mRNA composition [19]. This results in similar spot intensities for each gene both in the treatment and the control and will minimize any differences that could be caused by the different mRNA compositions from different samples. This spot-by-spot control correction can eliminate systematic errors that cannot be corrected with slide-wise normalization. Similarly, in the dye-swap design, two different dyes are used to label the same sample, which enables the correction of dye-gene interactions in the ANOVA model.

**Figure 8:**
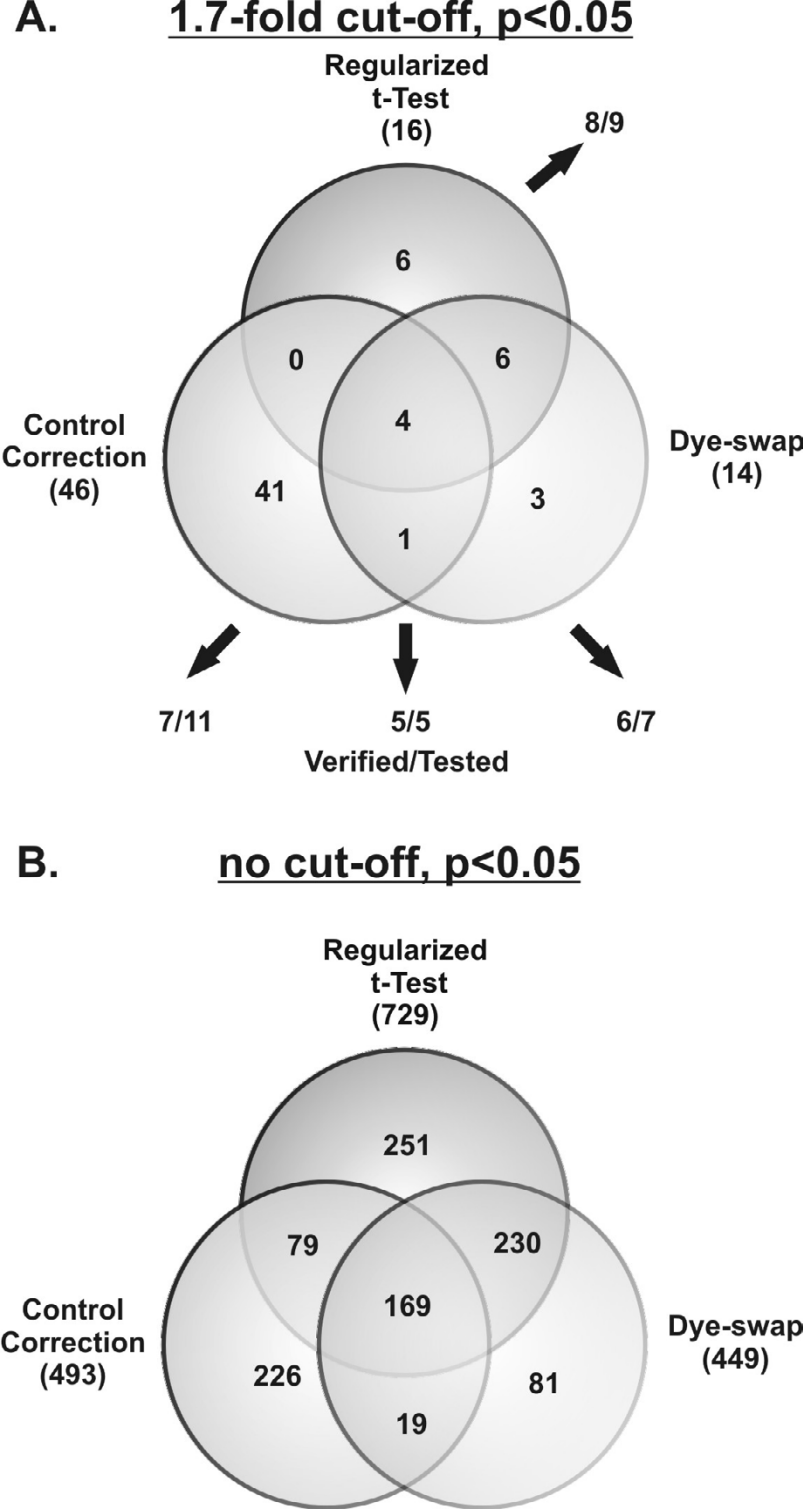
Summary of confirmation rates for the two methods. (**A**) A Venn diagram summarizes the number of genes identified by each experimental method using 1.7-fold and p < 0.05 cut-offs and the verification rate by northern blot. While all 5 genes common to both methods were confirmed, 7 out of 11 genes from control correction method were confirmed, and 6 out of 7 genes from the dye-swap method using the ANOVA were confirmed. 8 out of 9 genes identified with the regularized t-test were confirmed. (B) A Venn diagram summarizes the number of genes identified by each experimental method of p < 0.05 without a fold change restriction.

A summary of the results from this study are shown in the Venn diagrams in Fig. 8. Using the 1.7-fold and p < 0.05 cut-offs, the overall verification rate was 75% for the CCM and 92% for the dye-swap method using ANOVA. Among the 18 confirmed expression changes, all were below 3-fold and only six were above 2-fold. Many of the expression changes below 2-fold on the microarrays underestimated the fold-change measured by northern blotting. The accuracy was not dependent on microarray spot intensity as genes with both low and high signal intensities had similar verification rates (data not shown). The confirmation rates for both methods are comparable to methods reported by Mutch (87.5%) [27] and Tusher (92%) [28]. Of particular importance in this study is our high confirmation rates for genes differentially expressed by 2-fold or less.

The t-test used for the CCM and ANOVA for the dye-swap method depend on assumptions of Gaussian distributions that may or may not be present in a microarray experiment with a small number of replicates. Some efforts have been made to develop Bayesian frameworks that incorporate prior distributions in order to estimate the noise [24,29,30]. We therefore re-analyzed our dye-swap data using a "regularized" t-test [24]. Using this, we identified 16 genes that met our cut-off criteria, 10 of which were in common with the ANOVA analysis. Of those genes that we measured by northern blot analysis, 8/9 or 89% were verified. In summary, the regularized t-test revealed more genes than the ANOVA method with generally lower p values. If we eliminate the 1.7-fold cut-off, but maintain the p value <0.05, the CCM identified 493 genes, the ANOVA identified 499 genes, and the regularized t-test identified 729 differentially expressed genes (Fig. 8B). Among these, 399 were in common between the regularized t-test and ANOVA, 248 in common between the CCM and the regularized t-test, and 188 in common between the CCM and the ANOVA. These results demonstrate that if the false-positive rate remains the same, the regularized t-test is more sensitive than the traditional ANOVA and has extensive overlap, while the CCM has the least overlap between the other methods, but identifies different genes with slightly less specificity.

In our analysis, we selected genes based on their p values obtained from replicates of individual spots and did not adjust these p-values for multiple comparisons. This may be a major cause for the higher false positive rates for both of our experimental designs. For the CCM, if we apply Bonferroni correction, while we can eliminate all false positives, we would also miss a majority of the differentially expressed genes verified by Northern blotting. Therefore, if accuracy is the main purpose of a study, multiple comparison corrections should be used, while if sensitivity is the main purpose, then it should not be used with the understanding that the accuracy will be lower.

### Comparison of a dye-swap versus a control correction method experimental design

For our experimental design, the dye-swap method had a higher confirmation rate than the control correction method. This is, in part, due to the smaller variance that results from an effective doubling of the number of treated samples in the dye-swap method compared to the control correction method. Despite the higher degree of accuracy, the dye-swap design identified fewer genes and only detected down-regulated genes, whereas the control correction identified 3-times the number of genes that were both up- and down-regulated. However the control correction method was less specific for up-regulated genes. These differences may not solely reflect methodological differences, but likely result from experimental variability produced by performing the experiments independently on different days. Nonetheless, the results presented here suggest that both methods have clear merit in their abilities to show true gene expression changes, particularly for expression changes of 2-fold or less, and for genes with low signal intensities and/or low abundance.

The final decision as to which method is preferred depends on the experimental design. For example, the amount of sample and number of replicates required are important considerations both in terms of how difficult the RNA is to obtain and the number of samples that need to be compared. This also translates into the cost to perform the experiment. For instance, the dye-swap method generates a larger sample size for the same number of slides, thus producing greater significance when comparing gene expression between two samples. However this method requires a minimum of two slides and two different labeling reactions per sample. If the amount of sample is limited or population level replication is more desirable than individual sample replication, the control correction is more efficient since individual replicates for reverse dye labeling are not required and each sample can be run with only one slide. For example, to compare 6 treatment samples with a single control sample would require a minimum of 12 microarrays using the dye-swap method, whereas the minimum number of 8 arrays is possible using the control correction method; 6 for treatment samples and 2 for controls.

Another common experimental design used for time course or dose response studies is the reference design. In fact, the control correction method described here is essentially a modified reference design method where the zero time or dose point is the control-control comparison. As discussed above, using a very similar control sample to correct the series will give less false positives and negatives and a more accurate absolute value of the observed change than a dissimilar, pooled reference sample.

### Under-estimation of fold changes by cDNA microarrrays

Although our cDNA microarray results were accurate, the measured changes generally underestimated the actual changes measured by northern blots. Yuen et al. [6] similarly found that both oligonucleotide arrays (GeneChips by Affymetrix) and cDNA arrays underestimate fold changes compared to quantitative RT-PCR. The cause for this underestimation is not clear, however, it may be due to the limited dynamic range of dye signal or non-specific binding of the dye. Nonetheless, the limitations in accuracy and fold change estimation are far outweighed by the ability of microarrays to identify biologically important gene expression changes.

## Conclusions

This study demonstrated that dye-swap and control correction experimental design paradigms for cDNA microarray experiments are capable of detecting small, biologically important changes in gene expression with excellent fidelity while revealing important down-stream anti-proliferative effects of neuregulin on breast epithelial cells for future studies.

## Methods

### cDNA microarrays

Human cDNA glass microarrays, called the Alphamax Genechip, were obtained from Alphagene Inc. (Woburn, MA) containing 3333 cDNAs spotted in duplicate. The cDNAs used are identical to commercially available Micromax 2400 slides from Perkin Elmer Life Sciences (Boston, MA), most of which were derived from a human fetal brain cDNA library, with an additional 933 genes (gene list available upon request).

MCF10AT cell culture – MCF10AT cells were from Dr. Robert Pauley at the Karmanos Cancer Institute (Detroit, MI). The cells were cultured in DMEM/F12 media (Invitrogen) supplemented with 5% horse serum (Invitrogen), 10 mM HEPES buffer (Invitrogen), 10 μg/ml insulin (Sigma), 20 ng/ml EGF (Upstate Biotechnology), 100 ng/ml cholera enterotoxin (CalBiochem) and 0.5 μg/ml hydrocortisone (Sigma) at 37°C in 5% CO_2 _incubator.

Neuregulin treatment and RNA extraction – A recombinant human NRG β1 polypeptide (amino acids 14–246) was generously provided by AMGEN (Thousand Oaks, CA). After 3 days of culture, MCF10AT cells were treated with human recombinant neuregulin β1 form for 24 hours. MCF10AT cells grown under similar conditions without neuregulin treatment were used as a control. The cells were then harvested and total RNA was extracted using Ultraspec (Biotecx laboratories). The total RNA was cleaned up by Rneasy kit (Qiagen) and quantified using a fluorescent dye binding assay, Ribogreen (Molecular Probes). RNA purity was assessed by agarose gel electrophoresis. Proliferation assays were performed by counting quadruplicate cultures plated at 5000 cells/well using a hemocytometer.

Microarray hybridization – cDNA microarrays were used in a 2-step hybridization protocol that was optimized for the Genisphere dendrimer labeling method. Total RNA was reverse transcribed into cDNA containing a unique 5' primer tag, using the Genisphere 3DNA expression array detection kit. In brief, for each reaction, 3 μg of total RNA was reverse transcribed using 0.2 μM oligo-dT-Genisphere capture primer, 0.5 mM dNTP, 200 U Superscript II (Invitrogen) in 1X first strand Superscript II buffer at 42°C for 2 h. The RNA from the DNA/RNA hybrids was denatured with 0.5 M NaOH / 50 mM EDTA at 65°C for 10 min. The reaction was neutralized using 1 M Tris-HCl ph 7.5. The contents of the tube containing the NRG-treated and control cDNA were then mixed together and 3 μl linear acrylamide (Ambion) and 250 μl of 3 M ammonium acetate were added to them. cDNA was precipitated by adding 100% Ethanol and incubating at -20°C for 30 min. The cDNA was collected in a pellet by centrifugation at 13000 rpm for 15 min in a microcentrifuge. and resuspended in Alternate (formamide-containing) Hybridization buffer (Genisphere) at 65°C for 10 min and modified LNA blocker (Genisphere) with denatured Cot 1 DNA.

The entire mixture was added to the pre-hybridized array (Alphamax) for hybridization at 55°C for at 36 hr. A clear increase in signal was obtained with a 36 h hybridization compared to 16 h. After hybridization, the arrays were washed with 2X SSC and 0.2% SDS at 60°C for 15 min, followed by a wash with 2X SSC and another with 0.2X SSC at room temperature. For fluorescence detection, a second hybridization with the dendrimer was optimal. 2.5 μl each of the Cy3 and Cy5 dendrimer in Hybridization Buffer (Vial 6, Genisphere kit) were mixed with denatured Cot1 DNA and differential expander and the mixture was added to the pre-hybridized slides for hybridization at 60°C for 2 hrs. The slides were washed again as described above.

### Microarray data analysis method

#### Analysis of CCM experiment

Arrays were scanned with a GenePix 4000 A scanner (Axon Instruments, Inc., Union City, CA). Images were quantified using ImaGene Software (Biodiscovery, Inc. Marina del Rey, CA) that uses a local background subtracted from the signal. Signals not consistently detectable (background corrected signal lower than 2 times of background standard deviation) were eliminated.

We fitted loess curve to the log transformed data using the "loess" function in SAS software (SAS Institute Inc., NC) for intensity dependent normalization followed by a t-test to compare T/C with C/C ratio, gene by gene. The t-test was performed on the normalized log ratio with Welch correction for unequal variance. The control corrected fold change was calculated as:

log (fold) = log(T/C)-log(C/C)

#### Analysis of dye-swap experiment

For the dye-swap method we performed the same background correction and data filtering for absent genes and log transformations. We then used a two interconnect ANOVA model [22,23] and Mixed Model Analysis of Microarray Data (MANMADA)  to identify differentially expressed genes. First we use a normalization model for log-transformed intensity measurements:

*y*_*ij *_= μ + *A*_*i *_+ *D*_*j *_+ *AD*_*ij *_+ ε_*ij*_

Where μ is the sample mean, *A*_*i *_is the effect of *i*th array, *D*_*j *_is the effect of dye cy3 or cy5, *AD*_*ij *_is array dye interaction and ε_*ij *_is random error. The residue from normalization model is then used in following gene model to find treatment effects on each gene:

*r*_*ijkg *_= *A*_*ig *_+ *D*_*jg *_+ *T*_*kg*_

Where *r*_*ijkg *_is the residual of each gene from the normalization model, *T*_*kg *_is the treatment effect (control or treated), and *A*_*ig *_and *D*_*jg *_are the array and dye effects, respectively. The expression change for each gene is thus:

log (fold) = *T*_treated_-*T*_control_

### Northern blots

**5 **μg total RNA isolated from MCF10AT cells was run on a 1.3% Agarose/2.2M Formaldehyde gel as described previously [31]. Probes were prepared by PCR from the same clones used to spot the slides provided by Alphagene Inc except for AJ224442, X86779 and U62739, where clones BC011696, BI754516 and BG763631, with of over 99% identity, were used as substitutes. Probes were generated by random priming using PrimiT II kit (Stratagene) radiolabeled probes. The auto-radiographs within the linear range of the film were scanned with a flatbed scanner with transparency adapter and quantified using MetaMorph (Universal Imaging) analysis software as described previously [32]. For time course measurements, the amount of signal normalized for loading with either 18S RNA or GAPDH were plotted together after first setting 100% to the intensity of the control measurement at 48 hours and setting the lowest intensity value to 0%.

## Authors' contributions

BY analyzed microarray data. SR carried out microarray experiment. QL conducted MCF10AT cell culture and mRNA extraction. SA carried out northern blots experiments. RK provide input on microarray experiments. SD contributed ideas to data analysis. JAL conceived and design the experiment.

## Figures and Tables

**Table 1:** List of identified genes. Gene accession numbers, gene descriptions, fold-changes, and p-values for genes identified by the dye-swap method with ANOVA and regularized t-test analysis and the control correction method (CCM). Genes are broadly classified into three groups: proliferation-related, differentiation-related and unclassified.

**Proliferation-Related Genes**								
**Classification**	**Acc#**	**Gene Description**	**CCM fold**	**CCM p-value**	**Dye-Swap fold**	**Dye-Swap p-value**	**Regularized fold**	**Regularized p-value**

**Heat shock proteins**	NM_006597	heat shock 70 kDa protein 8	-2.8	9.5E-6	-2.9	4.2E-7	-2.9	8.4E-13
	M34664	heat shock 60 kDa protein 1 (chaperonin)			-1.9	3.9E-6	-1.9	4.8E-10
	L15189	heat shock 70 kDa protein 9B (mortalin-2)	-2.2	1.4E-3	-1.8	1.8E-6	-1.8	6.5E-6
	M22382	Human mitochondrial matrix protein P1			-1.8	1.5E-5	-1.7	2.9E-9
	M94859	Human calnexin mRNA, complete cds	-1.8	4.1E-3				
	L27706	Human chaperonin protein (Tcp20) gene complete cds	-2.1	3.8E-4				
**Transcription and translation**	D29677	helicase with zinc finger domain			-1.9	3.1E-5	-1.9	2.8E-10
	X91257	seryl-tRNA synthetase	-1.8	1.2E-3	-1.8	4.5E-8	-1.9	2.0E-11
	D30655	eukaryotic translation initiation factor 4A, isoform 2			-1.7	5.5E-5		
	U76111	Human translation repressor NAT1 mRNA, complete cds	-1.7	8.9E-3				
	M74719	transcription factor 4	-1.9	2.0E-3				
	X13293	Human mRNA for B-myb gene					1.7	4.2E-9
	U00968	Human SREBP-1 mRNA					-1.7	8.4E-9
	L41490	eukaryotic translation elongation factor 1 alpha 1-like 14	-1.8	1.4E-5	-1.7	5.3E-5	-1.8	5.0E-12
**Fatty acid and sugar metabolism**	D16481	Homo sapiens mRNA for mitochondrial 3-ketoacyl-CoA thiolase beta-subunit of trifunctional protein, complete cds			-1.8	5.6E-5		
	Y00711	Human mRNA for lactate dehydrogenase B (LDH-B)			-1.8	6.9E-5	-1.7	2.4E-8
	D78130	Homo sapiens mRNA for squalene epoxidase, complete cds			-1.9	1.2E-5	-1.8	9.5E-7
	M37154	Human glutamate dehydrogenase (GDH) mRNA, complete cds	-1.8	4.0E-3	-1.7	8.0E-4		
	U62961	3-oxoacid CoA transferase	-2.0	1.2E-4				
	Y13647	Homo sapiens mRNA for stearoyl-CoA desaturase	-1.9	1.5E-6				
**GTPase**	Y13286	Homo sapiens mRNA for GDP dissociation inhibitor beta			-1.8	1.8E-5	-1.7	1.3E-6
	X51408	Human mRNA for n-chimaerin	2.1	1.8E-2				
**Cell cycle**	U47413	Human cyclin G1 mRNA, complete cds			-1.7	9.6E-7		
	AF139897	Homo sapiens BASS1 (BASS1) mRNA, partial cds	-1.8	1.8E-3				
	D00265	Homo sapiens mRNA for cytochrome c, partial cds	-2.0	2.0E-2				
	X68836	H. sapiens mRNA for S-adenosylmethionine synthetase	-2.2	8.3E-6				
	U19251	Homo sapiens neuronal apoptosis inhibitory protein mRNA, complete cds	1.8	1.1E-2				
	S45630	alpha B-crystallin=Rosenthal fiber component [human, glioma cell line, mRNA, 691 nt]	2.1	2.7E-3				
	X86779	H. sapiens mRNA for FAST kinase	1.7	1.9E-2				
**Oncogenes**	X03541	Human mRNA of trk oncogene	-1.8	3.1E-3				
	M19722	Human fgr proto-oncogene encoded p55-c-fgr protein, complete cds	-1.9	5.0E-3				
	X77548	H. sapiens cDNA for RFG	-2.1	1.5E-3				
**Proliferation**	D63390	platelet-activating factor acetylhydrolase, isoform Ib, beta subunit 30 kDa	-1.8	4.4E-4				
	U62739	Human branched-chain amino acid aminotransferase (ECA40) mRNA, complete cds	1.7	1.2E-2				
	L12350	Human thrombospondin 2 (THBS2) mRNA, complete cds	1.9	2.2E-3				
	X06614	Human mRNA for receptor of retinoic acid	1.7	2.8E-2				
	Y07921	Human mRNA for serine protease					-1.7	1.1E-6
**Differentiation-Related Genes**								

**Tumor suppressor**	AF042857	Homo sapiens lung cancer antigen NY-LU-12 variant A mRNA, complete cds	2.1	4.4E-3				
**Differentiation**	M21300	Human small proline rich protein (sprI) mRNA, clone 15B	1.7	2.0E-4				
**ECM and vesicle trafficking**	M15395	Human leukocyte adhesion protein (LFA-1/Mac-1/p150,95 family) beta subunit mRNA	2.0	2.8E-2				
	S72869	H4(D10S170) = putative cytoskeletal protein [human, thyroid, mRNA, 3011 nt]	-1.9	3.2E-2				
	D21267	SNAP25 synaptosomal-associated protein, 25 kDa	1.9	1.7E-2				
	U95735	Human thrombospondin 2 (THBS2) mRNA, complete cds	-1.8	1.5E-5				
	X68194	H. sapiens h-Sp1 mRNA	-1.7	4.2E-2				
	Z74615	collagen, type I, alpha 1	2.5	5.3E-3				
**Immune-response**	U68030	chemokine (C-C motif) receptor 6	1.8	5.8E-3				
	X04701	Human mRNA for complement component C1r	-2.4	3.3E-2				
**DNA repair**	D79983	ring finger protein 144	1.7	2.3E-2				
	D29013	Homo sapiens mRNA for DNA polymerase beta, complete cds	1.8	7.3E-3				
**Unclassified Genes**								

	J02854	myosin, light polypeptide 9, regulatory	2.6	4.5E-2				
	X58141	Human mRNA for erythrocyte adducin alpha subunit	2.1	3.2E-2				
	AJ224442	Homo sapiens mRNA for putative methyltransferase	1.8	3.4E-3				
	X06661	Human mRNA for 27-kDa calbindin	2.1	9.0E-3				
	U24266	aldehyde dehydrogenase 4 family, member A1	1.7	2.5E-3				
	U62432	Human nicotinic acetylcholine receptor alpha3 subunit precursor, mRNA, complete cds	1.8	7.0E-3				
	U79259	Human clone 23945 mRNA, complete cds	-1.7	4.2E-3				
	M18533	Homo sapiens dystrophin (DMD) mRNA, complete cds	-2.5	1.1E-2				
	J05401	Human sarcomeric mitochondrial creatine kinase(MtCK) gene, complete cds	2.2	1.5E-2				
	D13315	Human mRNA for lactoyl glutathione lyase					-1.7	4.6E-7
	AB001740	Homo sapiens mRNA for p27					1.8	8.6E-4
	M15661	Human ribosomal protein mRNA					-1.7	1.3E-4
